# Space habitats for bioengineering and surgical repair: addressing the requirement for reconstructive and research tissues during deep-space missions

**DOI:** 10.1038/s41526-023-00266-3

**Published:** 2023-03-25

**Authors:** Alexandra Iordachescu, Neil Eisenstein, Gareth Appleby-Thomas

**Affiliations:** 1 School of Chemical Engineering, University of Birmingham, Edgbaston, Birmingham B15 2TT United Kingdom; 2 Consortium for organotypic research on ageing and microgravity, University of Birmingham, Edgbaston, Birmingham B15 2TT United Kingdom; 3 Healthcare Technologies Institute, University of Birmingham, Edgbaston, Birmingham B15 2TT United Kingdom; 4 Cranfield Defence and Security, Cranfield University, Defence Academy of the United Kingdom, Shrivenham, SN6 8LA United Kingdom

**Keywords:** Biomedical materials, Risk factors, Biogeochemistry, Biomedical engineering, Tissues

## Abstract

Numerous technical scenarios have been developed to facilitate a human return to the Moon, and as a testbed for a subsequent mission to Mars. Crews appointed with constructing and establishing planetary bases will require a superior level of physical ability to cope with the operational demands. However, the challenging environments of nearby planets (e.g. geological, atmospheric, gravitational conditions) as well as the lengthy journeys through microgravity, will lead to progressive tissue degradation and an increased susceptibility to injury. The isolation, distance and inability to evacuate in an emergency will require autonomous medical support, as well as a range of facilities and specialised equipment to repair tissue damage on-site. Here, we discuss the design requirements of such a facility, in the form of a habitat that would concomitantly allow tissue substitute production, maintenance and surgical implantation, with an emphasis on connective tissues. The requirements for the individual modules and their operation are identified. Several concepts are assessed, including the presence of adjacent wet lab and medical modules supporting the gradual implementation of regenerative biomaterials and acellular tissue substitutes, leading to eventual tissue grafts and, in subsequent decades, potential tissues/organ-like structures. The latter, currently in early phases of development, are assessed particularly for researching the effects of extreme conditions on representative analogues for astronaut health support. Technical solutions are discussed for bioengineering in an isolated planetary environment with hypogravity, from fluid-gel bath suspended manufacture to cryostorage, cell sourcing and on-site resource utilisation for laboratory infrastructure. Surgical considerations are also discussed.

## Introduction

Ongoing technological advancements and the recent launch of the first phase of Artemis I (on November 16^th^ 2022) are making a human return to the Moon and a first manned mission to Mars increasingly feasible. Detailed strategic plans have been created for the past 50 years by space agencies and governments (e.g. the Human Research Program at NASA^1^, ESA’s Space Resources Strategy^2^) for efficiently utilising outer space resources and establishing a human-operated lunar base as a testbed for an equivalent structure on Mars. This infrastructure is intended to be a global endeavour, where international partners can ensure the continuous and sustainable presence on the lunar surface whilst focusing on scientific and engineering objectives. Most recent plans (2022)^3^ detailed an integrated, multi-system approach to allow a campaign of human-led exploration on the Moon, and subsequently Mars, that also ensures a safe return to Earth. Within the transportation and habitation objectives announced by NASA in May 2022^3^ for generating a lunar infrastructure in preparation for a Mars settlement, the initial elements are focused on developing power grids that are evolvable to support continuous human and robotic exploration and that can be subsequently scaled-up to industrial levels. Ultimately, the aim is to develop the lunar infrastructure in terms of communications and architecture to support long-term science, advanced manufacturing capabilities and autonomy in construction, which is also considered at a large scale.

Importantly, amongst the key scientific targets^3^ are the establishment of a laboratory at the lunar South Pole, and undertaking a package of experiments in life sciences aimed at understanding the fundamental biological effects on human physiology and disease during short and long duration missions, as well as gaining new scientific information that could then guide system development. Finally, a requirement was identified for technologies that would monitor crew health and provide medical care in these environments, where emergency evacuation is not possible.

Therefore, the return to the Moon and the multi-part journey to Mars will require a significant number of resources, with the most important being undoubtedly the human aspect.

## Human missions - the physiological context

Humans offer significant advantages to robotic exploration, such as the ability to execute intricate tasks based on feedback, improvise and make choices and complex decisions. Importantly, they are also able to conduct a large number of scientific procedures which can provide further information on the long-term effects of microgravity on physiology and the exposure to an extra-terrestrial environment, including across generations.

At the same time, sending human crews to distant planets poses great physiological challenges which may become the biggest limiting factors in delivering a successful mission.

**Fig. 1:**
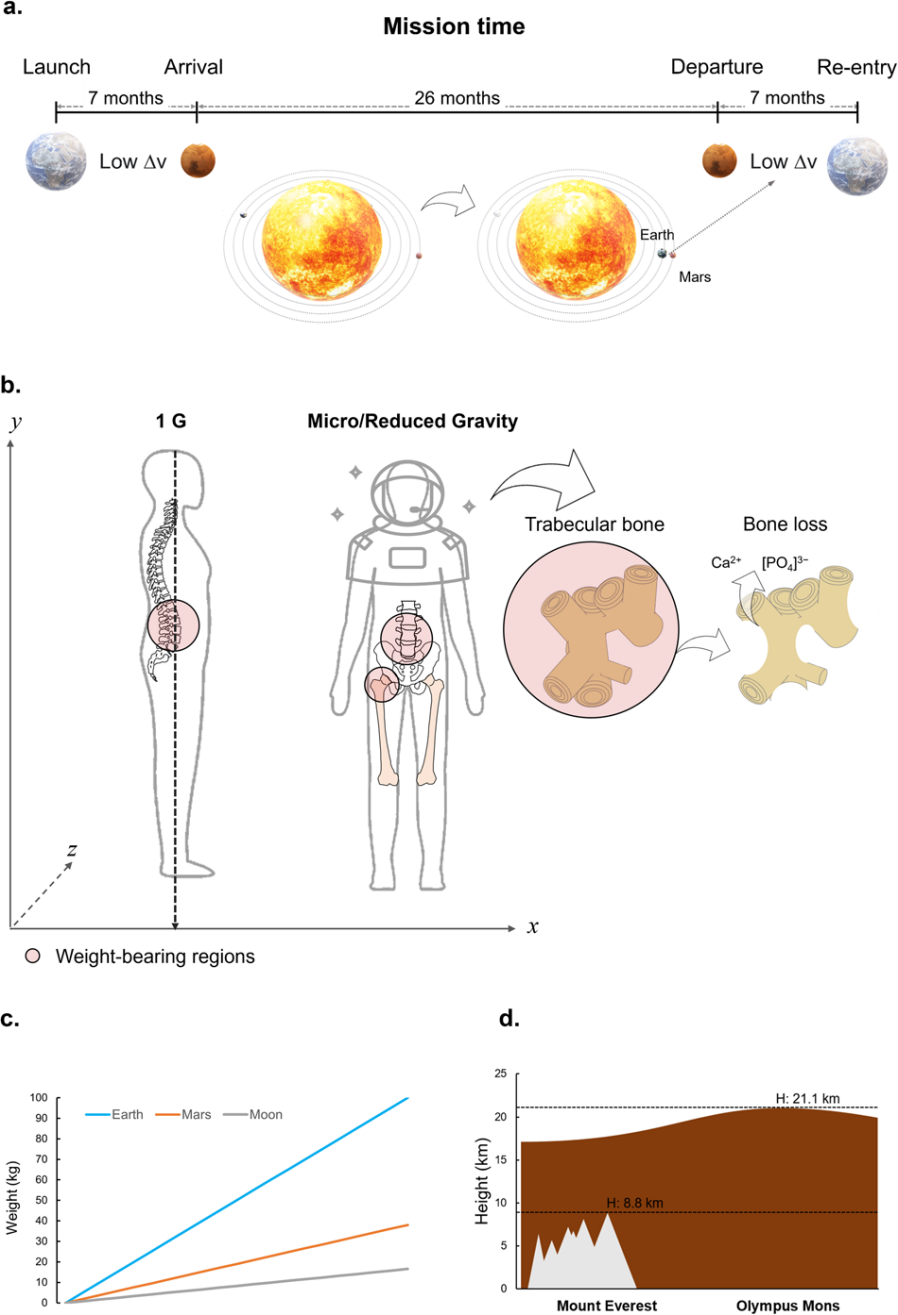
Physiological challenges on the skeletal system during deep-space missions. **a** An estimated timeline of a mission to Mars, involving approximately 7 months-long journeys between Earth and Mars, based on current technology. An extensive stay (approximately 26 months) on the planetary surface will likely be required as a minimum duration, while waiting for the Earth and Mars orbits to align in positions that will allow the most energy-efficient trip back. **b** Throughout the extended journey, the absence or reduction in the mechanical forces acting in the anatomical regions most adapted to withstand the gravitational force, such as the hip, femoral head, and lower back vertebrae, will lead to a significant loss of bone mineral in the form of Calcium and Phosphate deposits. **c** A range of relevant astronaut weights on Earth and the corresponding, reduced, weights in the gravitational environment of the Moon or Mars is presented. **d** Mars presents geographical features that are more challenging to navigate physically. For example, its highest mountain (Olympus Mons) is more than two times higher than Mount Everest. The lateral spine vector in **b** (1 G) was sourced^99^ and adapted under the CC-BY 4.0 license.

Human physiology is heavily adapted to the gravitational environment on Earth and the absence or reduction of this stimulus in low earth orbit (LEO) and beyond, on the Moon or Mars, can have profound effects even during short sojourns^4^. Some of the most immediate changes are in the hydrostatic pressure network formed by the cardiovascular system^5^ (caused by a shift of fluid to the upper part of the body), as well as a rapid reduction in tissue density and structure in muscular and skeletal sites^6,7^ adapted to counteract the gravitational force during standing or ambulation (Fig. 1). The microgravity-induced redistribution of fluid to the upper part of the body has profound effects on multiple organs. For example, it leads to an elevation of intracranial pressure, which increases to greater levels than intraocular pressure, leading to a pressure gradient that is thought to be responsible for a frequently reported visual impairment^8^. Simultaneously, the reduction in gravitational force leads to significant changes in the vestibular system, leading to impaired balance, locomotion, eye-head-hand coordination as well as motion sickness within the first days of spaceflight^9^.

Although astronaut candidates are extremely healthy individuals, space operations will require superior physical capabilities to aid with delivery of tasks such as extra-vehicular activities, installation and reparation of hardware and habitats. Furthermore, the cardiovascular-fluid changes may also impair the pharmacokinetics or pharmacodynamics of medications taken by astronauts^10^, including supplements, which means that these agents may not be effective or sufficient at mitigating tissue degradation, as well as raising further questions regarding dosage and safety^11^. Furthermore, the space environment poses additional issues in terms of radiation, a decrease in immune function and finally, confinement and isolation, all which have a combined effect on tissue health. In missions beyond the low-Earth orbit, radiation will be a significant challenge to manage, as crews will be exposed to several sources of energy, originating from solar particle events (SPEs, consisting of X-rays, gamma rays, and streams of protons and electrons), galactic cosmic rays (GCRs), which are high-energy protons and heavy ions from outside our solar system, and intravehicular secondary radiation^12^.The exposure of tissues to ionising radiation can lead to the formation of free radicals, DNA and oxidative damage in numerous tissues^11^. It has been estimated that for a 3-years long mission to Mars, crews would be exposed to whole body doses approximating 1-sievert (Sv) or more^13^. Therefore, significant shielding and countermeasures are required in order to prevent acute radiation sickness, degenerative diseases and to ensure crew survival and mission success.

In conclusion, the multitude of spaceflight-related changes will require an assessment of the extent of potential tissue damage and developing proportional countermeasures, such as enhancements in the life and health support systems. In the following sections of this article, we have placed a greater emphasis on considerations for the skeletal system and connective tissues, due to the operational scenarios that were considered, the available data on minor trauma throughout the US program, as well as the available technologies for prophylaxis and medical interventions in orthopaedics, which are likely to make the quickest progress in the basic and translational space applications.

## The impact on the skeletal system

The skeletal system is the most essential support structure in the body and maintaining its integrity is essential for performing any type of mechanical task. However, bones are some of the most affected and prone to damage organs during space missions. Bone tissue forms in response to mechanical demands and is heavily adapted at the tissue level from the nano to the anatomical scale to withstand compressive and tensile forces. In contrast, in microgravity, static and dynamic loading on this organ are absent due to the removal of external forces. Countermeasures^14,15^ designed to reintroduce these forces and mimic some of the ground reaction force, a factor that is key in bone stimulation, can only accommodate this need partially. However, a planetary environment, as opposed to the microgravity environment of LEO, might help in this respect. The presence of partial gravity on the Moon and Mars (1.62 m/s^2^ and 3.7207 m/s^2^ compared to Earth’s gravity of 9.807 m/s^2^, respectively)^16,17^ might help re-introduce this stimulus and slow down some of the tissue degradation. Therefore, during their stay, astronauts will experience approximately a sixth and a third of the Earth’s gravity. However, the lengthy journeys to these distant places will involve exposure to different phases of gravity and as such, different mechanisms of physiological re-stabilisation.

For example, whilst a journey to Mars would require approximately 7 months (Fig. 1a), long durations/waiting times will likely be spent on the planetary surface (a minimum of 26 months between optimal planetary alignments based on current technology, such as the one used for the Perseverance mission^18^) to allow for the most energy-efficient transfer between the two planets during the return journey (consisting of further 7 months in microgravity). Sustaining a presence on this planet will require even lengthier sojourns. The loss in tissue mass and structure during this time (Fig. 1b), is, based on current knowledge, likely to increase proportionally with longer durations spent in reduced gravity^19^. This will clinically manifest as a loss of tissue in the trabecular bone regions, increasing bone fragility and potentially compromising the ability to withstand re-entry forces on return. Although this tissue degradation may be slowed down by the presence of partial gravity, and in combination with adequate resistance exercise, it is possible that the loss of skeletal mass and demineralisation during the transfer period might increase the risk of damage shortly after landing on Mars. On arrival, setting up a base might require lifting and moving weights and equipment during construction - a representative range of weights experienced on Earth and the significantly different corresponding values in a Lunar or Martian gravitational field are presented in Fig. 1c. In addition, Mars has more challenging landscapes with geographical features that are harder to navigate. For example, the summit elevation of the highest Martian mountain (Olympus Mons)^20^ is more than twice that of mount Everest (Fig. 1d).

There are additional secondary issues associated with bone demineralisation, as the persistent release of calcium deposits from bone tissue into circulation can lead to its accumulation as kidney stones^21^, which can lead to further complications and the need for surgical intervention.

In addition, numerous studies suggest that the extensive exposure to galactic and solar radiation in deep-space environments will also contribute to bone and cartilage loss^11,22^, further increasing the fragility of these key tissues. Therefore, the effects of microgravity will likely be exacerbated by exposure to radiation in a long-term mission. Moreover, in rodent models, a simulated partial gravity scenario that mimicked the lunar environment (a sixth of normal Gravity) combined with low-dose, high-linear energy transfer irradiation still resulted in bone loss^23^, further highlighting the need for effective protective methods such as shielding materials and clothing.

Whilst a whole range of countermeasures such as exercise^14,15^, negative pressure application^24^ and dietary supplements^11^ has been incorporated in space missions over the past decades with varying levels of success, considerations about mitigating likely tissue damage on-site in general, for multiple organs prone to damage, might be equally important in short-term and would need to be assessed simultaneously. This is particularly important for clinical contexts such as tissue rupture and dislocation, bone fractures, skin burns, abrasion or lacerations, tendon/ligament tears, and blood loss. Some of the reported tissue damage situations in space have involved small traumatic injuries to both the skin and mucous membranes, for example during the NASA-Mir programme^25^, and by 2009, 219 in-flight musculoskeletal injuries were identified throughout the U.S space program, most involving hand injuries during translation between modules, resistive exercise and extra-vehicular activity components^26^. However, major trauma is a strong possibility during deep-space exploration and long-duration spaceflight^27^.

## Regenerative medicine research in space applications

Most recently, the importance of regenerative medicine for long-term missions and the need to create metabolically-functional (and vascularised) human tissue in a controlled (in vitro) environment was most significantly approached by NASA with challenges such as the Centennial Vascular Challenge^28^ and the installation of 3D bioprinting facilities in low Earth orbit through the ISS National Laboratory^29^ which has allowed the research of stem cells^30^, spheroids (i.e. spherical agglomerates of cells)^31^, organs-on-chips^32^ and implementation of biomanufacturing (bioprinting)^33,34^, albeit at the current level of development, with the limitations currently posed by printing biological structures.

Furthermore, the Tissue Chips in Space programme, a partnership between the ISS National Lab and the National Center for Advancing Translational Sciences at the National Institutes of Health (NIH), which commenced in 2016, allowed the extensive use of microfabrication advances to create organ-on-a-chip platforms that could be studied on board the ISS for translational purposes, testing therapeutics and for gathering information on human physiology and disease in the extreme environment of space^32^. The projects conducted varied from studying the blood-brain barrier, immunosenescence, lung infection, cardiac dysfunction, post-traumatic osteoarthritis, proteinuria and kidney stones, inflammation in the intestine, sarcopenia and engineering heart tissue^32^, thus accelerating efforts to understand the effects of the spaceflight environment on multiple organs and systems. In parallel, through the NASA Space Biology Program (2023–2026) these types of studies will also be accompanied by projects using small invertebrate and vertebrate organisms as models for different human physiological systems, to provide further information on acclimation and adaptation to the many spaceflight stressors and thus help towards supporting organisms to thrive in deep space^35^.

Simultaneously, the modular space station environment of the ISS permitted numerous habitation studies by becoming the home of hundreds of astronauts, a laboratory and ultimately a validated proof of concept for long-term tissue and cell experiments in space. In addition, it allowed the generation of extensive knowledge on technical systems for life support and scientific procedures, radiation protection, environmental monitoring, crew health and countermeasures (e.g. exercise, diagnostic and medical equipment) and the long-term food storage^36^. Therefore, this knowledge can be now be used for estimating the requirements of habitats further than LEO, which can be performed in phases and can integrate analogue and ground-based knowledge as well.

## Medical options on-site - from telemedicine to autonomous medical support

Habitats further than the low-Earth orbit, such as deep-space habitation systems will not likely benefit from receiving supplies or assistance from Earth in emergency-type situations. For example, the distance between Earth and Mars ranges between 54.6 million kilometres to approximately 200 million kilometres. In contrast to the Moon-Earth communication delay, which is in the order of seconds, there is a significant communication latency between Mars and Earth^37^, that can range between 5–20 min depending on planetary positions. Secondly, relaying/receiving information can require high data rates and in addition, it is unlikely that crews can rely on intermittent communications in those situations where expert medical support will be required immediately. These limitations mean that telemedicine, remote medical support and terrestrial assistance to the crew in real-time might not always be possible during complex medical procedures and could be further compromised by a potential equipment failure. Therefore, an operational shift is required towards autonomous medical activities, a challenge which requires careful considerations to ensure an adequate medical infrastructure at these sites. These include considerations regarding crew professional and mission-specific training during selection. In LEO (ISS), The Crew Medical Officer (CMO) was not necessarily a physician, and had received medical training instead^27^, due to the ability to evacuate in case of an emergency. However, a general surgical specialist will be required to deliver complex interventions following severe incidents beyond LEO, while also coordinating with remote medical teams via telemedicine. Additionally, it might be necessary to incorporate robotic surgery as a supporting measure where medical crew selection will be limited. Ultimately, these aspects will guide habitat design, as well as the technical infrastructure required in both short and long-term sojourns. Therefore, it will be essential that the facilities to mitigate damage, particularly significant trauma or injury, are present on-site. Under normal clinical circumstances, tissue reconstruction or replacement is performed using a combination of medical and bioengineered solutions, which range from metallic implants and deproteinised xenografts to tissue engineered autologous cell-containing matrices, which can be customised to replace the deteriorated tissue.

## Building a deep-space biomedical habitat to address the physiological challenges

Crews will require access to a sustainable source of tissue substitutes, biochemical scaffolds, haemostatic agents, or biomaterials such as dental fillers in addition to specialised medical-surgical training in order to address a wide range of health challenges, from minor tears to serious emergencies. With longer sojourns, these will be needed in additional operational scenarios, such as a lack in response to countermeasures, the potential absence of adequate nutrition, or the likely failure of equipment. In addition, healing in connective tissue is known to be impaired in microgravity, as well as the response to growth factors, which suggests that spaceflight might impair the capacity of wounds to respond to exogenous stimuli^38^.

The need for a quick recovery of function means that some form of tissue replacement/analogue would have to be developed on-site in useful time, implanted or applied to the injured site shortly after, or incubated (in the case of biological implants) as per typical procedures until a desired morphology/maturation stage is achieved. The potential for a significant interval before astronaut tissue function is restored feeds into the wider issue of crew numbers, as well as redundancy in training and capabilities. The repair and reconstruction process will, in turn, require specialised equipment and simultaneous considerations that have to match safety and efficiency with sustainability and practicality from a space hardware perspective. In addition, these biological-surgical-rehabilitation facilities would have to be located in proximity to each other and constructed as a contained, multi-module habitat due to the requirements of both wet lab and surgical facilities to ensure sterility and biohazard containment, and the likelihood of complications arising during a recovery period post-implantation (e.g. infections, the need for further intervention).

Therefore, estimating the requirements of a tissue substitute production module during deep-space missions, as well as the concomitant clinical requirements to support operations, is essential for a long-term sojourn and establishing a settlement on a different planetary surface. It is important to generate a set of logical predictions on the requirements of this specialised biomedical habitat containing both tissue development and repair facilities, which will be essential for supporting human health and performance on other planets. Such a habitat would be beneficial not only for mitigating the impact of the space environment on human physiology, but also for conducting personalised research into astronaut health using both microphysiological systems, such as organoids, organotypic cultures, and organs-on-a-chip; but also, with time, multi-tissue constructs and eventually organ analogues, providing further information on long-term planetary colonisation.

## Habitat design considerations

### Energy and advanced life support

**Fig. 2:**
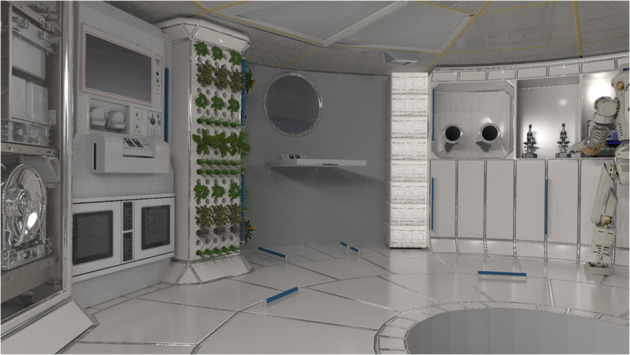
Supporting habitation and research in deep-space. A module within a deep-space habitat, containing a plant growth wall, essential for conversion of carbon dioxide to oxygen, robotic assistance, scientific kit (microscopes) and a glovebox to allow the contained manipulation of objects. Image credit: NASA^36^.

A biomedical enclosure in deep-space would have to ensure a high degree of autonomy and sustainability and be adequate firstly in terms of environmental support (atmospheric, pressure, acoustic, microbial contamination, radiation protection) and sustaining human presence (air purification, water recovery, waste processing). Figure 2 presents an artistic interpretation developed by NASA of a module within an envisaged deep-space habitat, containing a plant growth wall, essential for conversion of carbon dioxide to oxygen, robotic assistance, scientific kit (microscopes) and a glovebox to allow the contained manipulation of objects^36^.

Secondly, the habitat would have to be autonomous in meeting its energy/electricity demands and allowing stable communication networks with inter and extra-planetary bases. It also needs to integrate specialised requirements such as enhanced air filtration and plumbing to support tissue work and biohazardous waste processing.

Additionally, planet-specific considerations exist, such as positioning on the planetary body and timing of operations to match a range of seasonal phenomena. Although lunar missions will be ideal for identifying tasks which will help to reduce Earth dependence, the strikingly different environmental conditions between the Moon and Mars might require substantially different considerations. These differences include the available resources on-site, gravitational conditions, atmosphere presence/composition and mean surface temperature. Solar panel power generation will be key, although the energy storage capacities might be limited in early stages. Furthermore, Mars is particularly abundant in resources relevant for building a biomedical habitat and therefore sending technologies for energy conversion on-site will be key in early stages.

## Modular set-up of the biomedical habitat

The hostile environments of the Moon and Mars requires that the modules of this habitat are located in close proximity to each other. For example, is it critical that that the surgical module of the habitat is located in close proximity to the tissue engineering laboratory, to prevent delays, damage from transportation due to the challenging environmental and geological conditions and to minimise the life-support resources required during transportation of viable tissue. Simultaneously, a nearby recovery module is necessary to prevent the recipient moving during the early phases of tissue regeneration and therefore risk implant failure or non-integration.

**Fig. 3:**
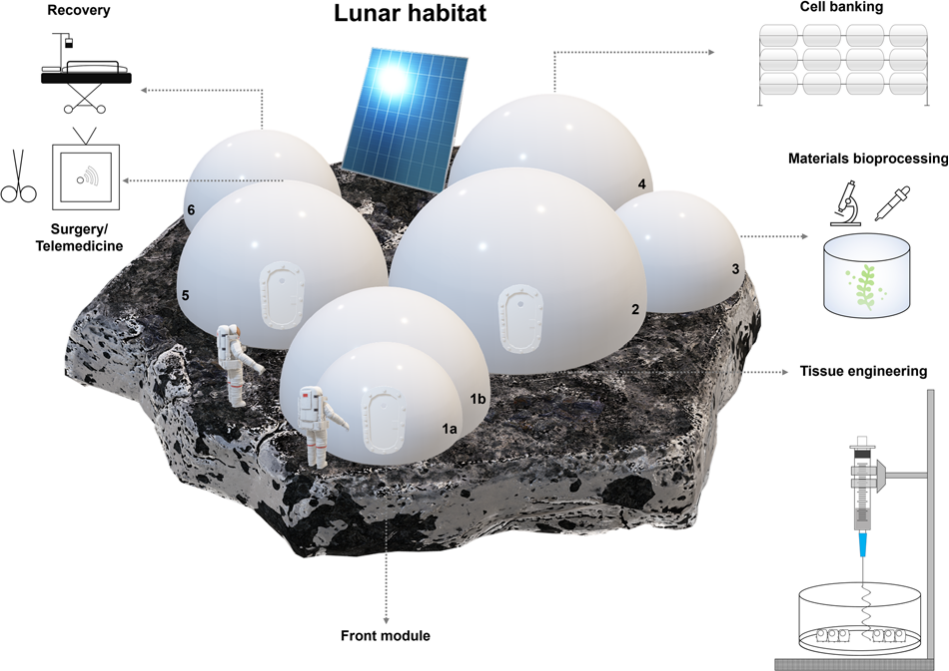
Structure of a biomedical habitat prototype supporting the production of tissue substitutes and medical procedures on the Lunar surface. Accompanied by solar panels to meet energy demands, the habitat would contain 6 modules to support these activities. Three modules would be required for engineering and expanding tissue analogues (M1–3), module M5 would be used for surgery and M6 would be used for recovery. The tissue/bio- engineering module (M2) containing advanced biofabrication facilities and bioreactors, would be supported by two auxiliary modules for biomaterial processing and extraction (M3), and a separate cell banking module (M4) to minimise traffic through this module. Access to either the medical branch or the bioengineering branch would be performed through module M1, containing two separate units (1a and b) containing an airlock (1a), and a sterilisation chamber(1b) to allow the transition from the dusty outside surface into the sterile environment and prevent striking changes in pressure when entering the main habitat. Access from the tissue engineering (M2) to the surgical module (M5) is possible, whilst the recovery module is only connected to the surgical module.

Based on these operational considerations, a minimum number of 6 modules would be required to support the activities. Figure 3 presents a possible set-up that would be suitable for construction on the Lunar surface. Accompanied by solar panels for energy generation and powering specialised equipment, three modules would be required for engineering and expanding tissues (M1–3), while module M5 would be used for surgery and M6 for recovery following an operation. The tissue/bio-engineering module (M2) containing the main array of kits, from bioreactors and incubators to bioprinters, would be supported by two auxiliary modules where activities such as biomaterial processing and extraction could take place on-site (M3), whilst module M4 would help separate and contain cell biobanking so that traffic is removed through this module. In this design, access to either the medical branch or the bioengineering branch would be performed through module M1, containing two separate units (1a and b) including an egress/ingress airlock (1a) to prevent striking changes in pressure when entering the main habitat and a sterilisation chamber in 1b to allow the gradual transition from the outside environment into the sterile environment of the laboratory or surgical module. Access from the tissue engineering (M2) to the surgical module (M5) would be possible and ideal, whilst the recovery module would only be connected to the surgical module.

## Protection against radiation and cosmic objects

**Fig. 4:**
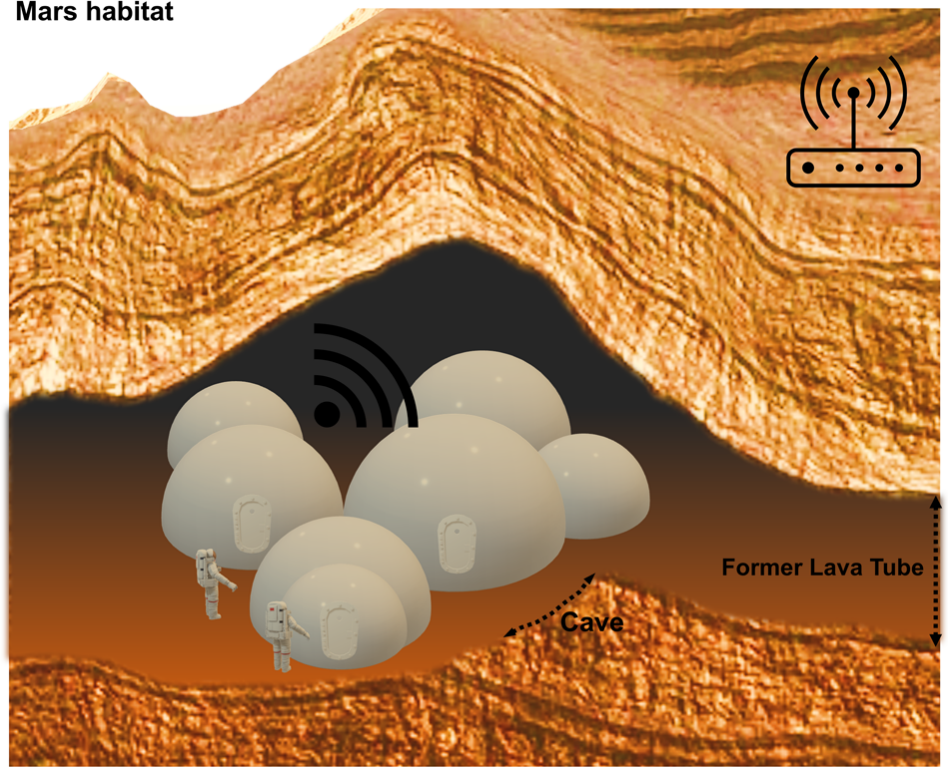
A biomedical habitat positioned inside the cavernal-lava tube sites on Mars. An interesting concept has been previously suggested, that would make use of already existing caves and former lava tubes within the Martian underground, in order to provide protection against radiation and falling cosmic objects. This positioning might be particularly useful for this type of habitat, as it could facilitate construction, containment and maintaining atmospheric conditions. Internet-like communication capabilities, connected to surface nodes, would aid with data exchange and video-audio communications.

One of the main challenges with the construction of such a critical habitat at a planetary surface is the exposure to significant cosmic radiation^39^, as well as falling micro-meteorites or even larger objects, which can significantly damage key structures. In other planetary environments, such as Mars, additional geological environments may be a likely option for shielding. A possibility has been brought forward previously that would make use of Lunar caverns or extensive Martian underground pyroducts for this purpose. (Fig. 4). Mars, for example, contains many extensive caves and lava tubes that formed following previous volcanic activity and are located close to extinct volcanoes^40^. A comparative assessment of size and morphology indicated that the equivalent tubes on the Moon and Mars are up to 3 orders of magnitude more voluminous than the equivalent structures on Earth^41^, therefore allowing habitat structures. This positioning would be particularly useful for this type of habitat, offering further advantages, as the cavern walls would be useful for construction, containment and maintaining atmospheric conditions^42^.

Recently, data from the Diviner instrument aboard the Lunar Reconnaissance Orbiter indicated that the lunar pits and caves (assessed in the Mare Tranquillitatis region) are also sites with greater thermal stability compared to the dramatic fluctuations at the surface, with minimal variability throughout the lunar day, and harbouring comfortable temperatures of approximately 17 °C (63 °F) compared to the surface (which can reach approximately 124 °C during the day and −178 °C before sunrise)^43^, making the subsurface environment more hospitable^44^.

To allow voice and video communications, control of robotic instruments and exchanging data, the habitats placed within superficial or deep subsurface locations will have to be linked to the surface communication systems and it is likely that this infrastructure will be based heavily around internet-like type connections, that would provide a high-speed communication network, something that is already being explored by the space agencies. For example, the NASA Space Communications and Navigation program (LunaNet) is planning to offer a network approach similar to internet, through the installation of multiple nodes connected with others on and around the moon^45^.

### Bioengineering module requirements

Bioengineering, in simple terms, involves the development of biochemically and histologically similar analogues (or ‘artificial tissues’) using cells, polymeric matrices, growth factors and bioreactors. Cell-containing analogues can be cultured in controlled environmental conditions, allowing tissue-like growth and function. As an area, bio/tissue engineering was developed to address organ failure and the current shortage of donor tissue experienced worldwide, to screen novel compounds, as well as for personalised medical research. These tissue analogues can be conveniently cultured using artificial systems (e.g. incubators providing adequate physiological conditions) and ultimately be applied to repair damaged sites, some technologies which are already available on the market^46^, particularly for cartilage and skin repair, and are discussed later in this article (see (Bio)Medical scenarios and prioritisation of activities).

The concept of growing cell-seeded-matrices for tissue engineering within a space enclosure was first shaped as early as 1990s^47,48^. For example, some of the earliest studies, performed on the Mir Space Station, combined cartilage chondrocytes and biodegradable polyglycolic acid (PGA) scaffolds to generate three-dimensional tissue^47^. Cartilage was a particularly useful tissue to test in microgravity due to its robustness and significantly lower metabolic requirements compared to other tissues. Over the subsequent decades, multiple tissue engineered models from several tissue types were flown to the low Earth orbit, which would provide further information on genetic and molecular responses in microgravity^49^.

With the establishment of the International Space Station National Laboratory, the concept of tissue and organ bioengineering in microgravity was further shaped, with the scope of using the ISS habitat as a platform to identify research questions and potential challenges to enable the next generation spaceflight regenerative medicine research^50^.

Bioengineering and implantology rely heavily on biochemically-relevant organic and inorganic matrices which can be inserted at the site of tissue damage to allow geometrical repair and restoring function. Where living tissue is harvested, either as autologous grafts (from the same patient), isograft (from a genetically identical individual), allograft (from a generically different individual) or xenograft (from an animal source)^51^, their handling requires a number of highly-sensitive steps which are both time and storage-dependent. A priority is preserving viability of the structure during transfer and maximising sample integrity through minimal processing, to ensure a successful implant integration. In the case of autologous grafts however, surgical removal may lead to tissue damage/necrosis at the site where the excision was performed, which risks creating further complications and the need for additional monitoring and interventions.

Within a space exploration context, during a potential tissue trauma or injury event it is likely that the available sources of tissue will be limited to these autografts. This is because donation of tissue from additional crewmembers would pose further challenges firstly from an ethical perspective, and also regarding biocompatibility, as there is a risk of immuno-rejection and a need for the recipient to receive immuno-suppressant medication to combat the response to a foreign tissue. It is difficult to estimate the additional complications posed by the environmental space conditions on this process, as the extra-terrestrial environment is known to lead to an undesired immunosuppressive action^52–54^.Therefore this interaction would need to be addressed in future research. Further, introducing additional risks to affected crew members might pose an unacceptable challenge to mission continuity/success.

This means that a large proportion of the reconstructive options in an injury scenario (particularly in orthopaedic interventions e.g. ligament, tendons, cartilage and bone damage) would initially involve those based on well-characterised biochemically and structurally-relevant matrices (polymers, ceramics like TCP, metal alloys and composites). In the case of bone tissue degradation, additional, physico-chemically-treated xenografts (e.g. biological hydroxyapatite) similar to those currently used in clinical practice would allow restoration of structure, function and some of the local biochemistry in the short-term. These could be carried in sterile-packed containers and would not require any special storage conditions. These could be used in addition to medical stabilisation materials such as skeletal screws and sutures. Some of the traditional implant materials (e.g. metallic alloys), although not ideal from a from a biochemical perspective, will likely be essential in short term to restore mechanical support, anatomical structure, function and therefore mobility.

Within the timeframe of the lunar mission (late 2020s–early 2030s), it is likely that simplified tissue engineering approaches (e.g. extracellular matrix-type biomaterials) can provide viable solutions for astronauts, such as injectable hyaluronic acid injections for cartilage repair in knee degeneration, which can be followed by more complex interventions in subsequent decades (2040s–2050s), such as total knee replacement (Fig. 8). There are additional, acellular, extracellular matrix-derived materials and natural polymers with more native biochemistries to in vivo tissues that could offer promising solutions for the repair of multiple types of tissue in the longer term. These include collagens^55^(e.g. type I or II), fibrin^56^ and laminin^57^, chondroitin sulfate^58^, heparan sulfate^59^ and many others.

These substrates could be implanted on their own or coated with bioactive factors to accelerate the integration with native organ structures over several months. These could also be more effectively used in later years in combination with primary cells expanded from small tissue samples from the affected crewmember, which would aid with a quicker restoration of function.

**Fig. 5:**
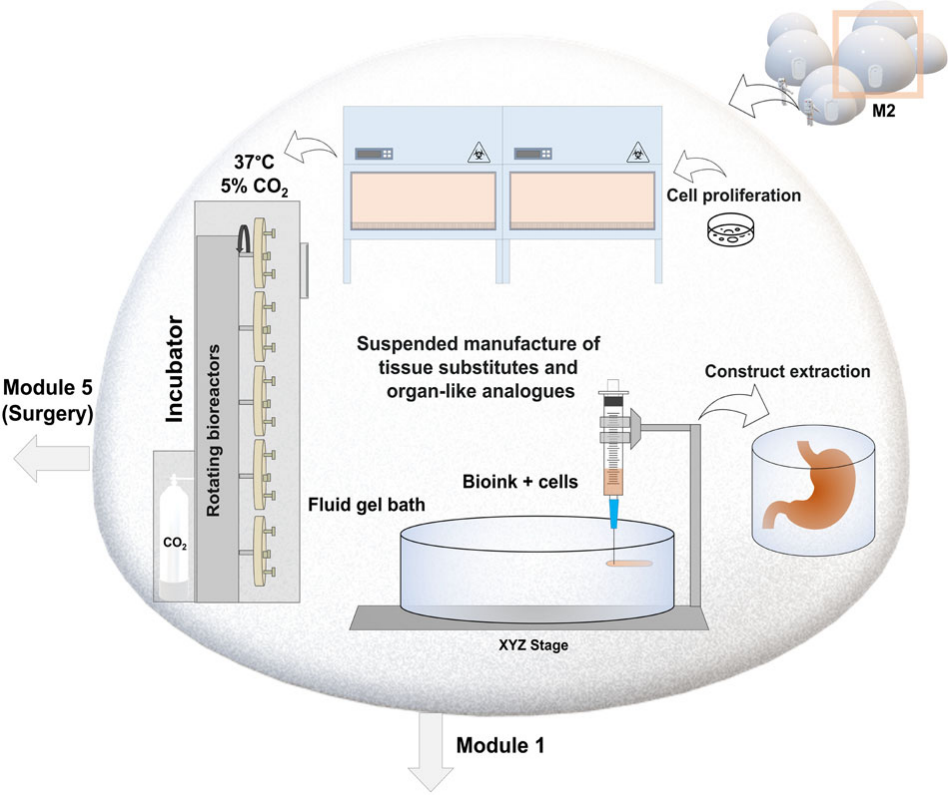
Operations within the tissue/bio-engineering module. This module of the habitat (M2) would host specialised equipment for cell proliferation, tissue engineering and research, rotating bioreactors and advanced biofabrication facilities, including sterile/contained 3D bioprinting devices, which would help generating constructs of relevant geometries and further organisation (both cellular and acellular). Bioprinting of tissue-like prototypes and components in a reduced gravitational planetary environment can be facilitated through suspension in support matrices (fluid gel baths) produced in module M3.

In the case of biological implants developed with cells, the relevant population of cells could be isolated from the crewmember following a clinical event and cultured using incubation facilities present at the site within the tissue engineering module (Figs. 3–5). These autologous tissue-engineered constructs developed ex-vivo would present a minimal risk of immuno-rejection, as the raw components (cells, inorganic matrices and even the organic matrices) used for generating constructs could be isolated from the crewmember. For example, fibrin, a haemostatic agent, can be recreated from the normal human blood components fibrinogen and thrombin^60^, subsequently mixed with inorganic (bone hydroxyapatite) matrices and osteoprogenitor cells, generating personalised bone tissue constructs (some relevant technologies currently available on the market are described later in the article).

In this context, the European Space Agency, in partnership with OHB System AG, has already built and launched to the ISS^61,62^ a handheld portable device that could serve this purpose by delivering cells within bioinks to a wounded site. This technology, named the BioPrint FirstAid device (essentially a handheld bioprinter), was trialled between October 2021 and September 2022, and is intended to deliver a patch or ‘biological plaster using the astronaut’s cells. This could accelerate the healing process and was designed for use specifically in these types of settings, such as an isolated habitat. Advantageously, the device was designed to be mechanically-driven, which removes the need for power supply. The main parts consisted of a dosing device in the handle, an ink cartridge, a print head and support wheels to assist with application -an accessible feature that would help with use by non-medically trained staff. This prototype has not yet been tested with cells; instead, fluorescent markers were used as substitutes for this component to date.

Alternatively, a ‘bank’ of relevant cell samples could be prepared before the mission from donated tissue from each crewmember and carried as cryovials of differentiated cells (containing 500k-1 million cells from each tissue) and expanded when needed. Exhausted vials could be replaced during the mission if medically feasible. This bank would have to be separated to an auxiliary module to reduce traffic from other operations (Fig. 3). It is important to note, however, that production of a sufficient number of cells to cellularize large structures such as a large tissue section or an organ is currently a major challenge. The number of mammalian cells in the body is estimated to be in the trillion range, totalling approximately 30 × 10^12^ for a 70 kg male and 21 × 10^12^ for a 63 kg female^63^. This varies across organs, as muscle and connective tissue cells such as myocytes and adipocytes are thought to constitute a smaller fraction (0.2%) of this number (value estimated for a reference adult male)^63^. As such, many of the initial, engineered tissues would rely on cell proliferation at the site of implantation. In subsequent decades (e.g. 2040s–2050s+), the implementation of industrial-level stirred tank or roller bottle bioreactors might help with producing cell numbers at a larger scale (e.g. 10–20 m^3^ or larger vessels)^64^ for this application. In addition, if stem cells are to be used as an alternative source to primary cells, this would likely require a significant number of frozen supplements, growth and signalling factors to tune these cell populations into the relevant tissue types.

In the short-term, these procedures would likely focus on the generation of basic personalised tissues which can range from constructs that can be applied on the skin surface to treat cuts, burns and other lesions to orthopaedic constructs that can be implanted to augment bone fractures or degradation; and subsequently, with further developments in the following decades, composite multi-tissue constructs which can also be vascularised. In contrast, the initial phases of damage mitigation and implant integration will most certainly rely on cell invasion and re-vascularisation from local tissue networks. A timescale of these incremental stages and their implementation is presented in Fig. 8.

A second target for this module would also be developing matrices that can deliver therapeutics at the site of injury through minimally invasive methods such as skin patch application or injection, which would be less invasive and require less training/skills from a medical perspective. The administration of such compounds is essential also to minimise the off-target accumulation of agents, due to changes in fluid distribution in reduced gravity, as discussed previously.

The tissue engineering module could also host further biofabrication facilities, including sterile/contained 3D bioprinting devices, which would help generating constructs of relevant geometries and further organisation (Fig. 5). This is necessary in the short-term also because a further operational objective of this module would be conducting personalised research into the physiological response and adaptations to the planetary environment using tissue avatars. Tissue-engineered constructs can also be applied to investigate preventative and therapeutic countermeasures, by conducting personalised research into astronaut health and studying the alterations in cells, tissues, genetic and molecular activity induced by the environmental conditions. For example, they may become a useful tool for studying the effects of radiation, pharmacokinetics, wound healing or screening loading and nutritional regimens to enhance tissue repair or prevent degeneration. Similarly, much larger and complex structures, such as multi-tissue prototypes, and early versions of organ analogues, which are many decades away from full development and operational-level technological readiness, will be essential in early stages not necessarily for applications in regenerative medicine, but as complex research platforms for conducting basic and translational investigations into the effects of environmental conditions on tissue architecture and organ functions. These true-scale analogues would facilitate the integration of a range of markers, reporters and sensors, which could provide information regarding protein and gene activity as well as histological changes. While representing simplified versions of the in vivo structures, they can recapitulate many aspects of tissue architecture and biochemistry, which is ideal for bioengineering research and could potentially be implemented within the required lunar and Mars mission timeframes.

Finally, the interplay between surgery and tissue development (in terms of the order of procedures and multiple steps required) is another rationale for building these different modules within the same habitat. For example, minor tissue explants required for cell isolation, proliferation and incubation within matrices would need to be transferred from a surgical module to the tissue engineering module, whereas mature/ autologous grafts developed in the latter facilities would need to be subsequently transferred to the operating theatre for implantation.

## Cell culture, handling and incubation

Our experience with wet lab operations outside of Earth developed with increased scientific activities on the ISS to study fluid dynamics, high-throughput biological processes, DNA sequencing and microbiological alterations in microgravity, which also facilitated the optimisation of these processes in terms of handling, efficiency and safety^65^. Many fluid-related operations key to cellular culture and tissue engineering, such as sample preparation and extraction, pipetting (injecting, ejecting, aspirating), the use of standardised microplates, handling of a large number of samples, mixing and sterile containment, have been optimised during recent programmes^65^. This involved surface and geometrical modifications of typical laboratory plates (e.g. custom 3D printed capillary fluidic wells) and the addition of fan/flow assisted droplet capture, which allowed the improvement in handling of biological samples dependent on liquid suspension in the absence of gravity and greater fluid control. This is relevant for handling many key components of tissue engineering, such as cells, microorganisms, enzymes, matrix proteins and polymeric matrices. In addition, human cells and organotypic structures can be cultured at a larger scale in microgravity owing to the development of rotary vessel bioreactors which can provide a continuous suspension and perfusion^48,66^.

## Automation of tissue and future organ-like analogues production

It is likely that the tissue substitutes produced initially within a tissue engineering module (both acellular and cellular) will use a combination of manual and semi-automated methods. Similarly, many surgical reconstruction steps rely heavily on manual procedures, which require the surgeon to mix the implant material with a suitable delivery agent and apply it to the affected site.

Additive manufacturing is considered an essential enabling technology for in-space manufacturing, allowing the production of needed parts in useful time, from electronics to habitat parts to engineered tissues.

Biofabrication, combining additive manufacturing with bioassembly, is thought to offer a promising solution for printing transplantable tissues in the long-term, as it can generate tissue-like structures in a semi-automated manner using cells suspended in suitable bioinks. The ability to use multi-axis robotic arms to produce biological structures from 3D coded files (and hence patient-specific anatomical designs) offers the promise to produce structures of unmatched complexities.

The application of bioprinting in microgravity is also a fast-evolving area of research and its current limitations as a technology for space exploration have been discussed recently^67^.

The major advantages of bioprinting compared to traditional tissue engineering are perhaps that the required constructs could be manufactured in a personalised shape and size, can introduce structures such as pores in a controllable fashion; involve multiple biomaterials and integrate multiple types of additive layer manufacturing technologies into one system. It is important to stress, however, that printing of suitable matrices and cells in a suitable conformation does not automatically generate a functional tissue and multiple culture stages are required post-printing to allow the cell component to develop the native behaviours found in vivo.

There are multiple types of bioprinting technologies available, including inkjet-based^68^, extrusion-based^69^, laser assisted^70^ and stereolithographic (light-curing)^71^. Some can include or avoid the use of scaffolds as temporary templates (e.g. microcarriers, polymers), and each presents multiple advantages and limitations in generating the native tissue architecture. For example, some types of printing are more successful in achieving a suitable resolution at the nano-micron scale compared to other types, while some are constrained by the extrusion conditions such as nozzle impedance and small dispensing volumes, which can increase processing time and reduce cell viability^72^. There are simultaneous challenges with bioink formulation and optimisation, to match biocompatibility with their performance through the system. Specifically, bioinks, which can be naturally-derived or synthetic hydrogels, must have shear-thinning fluid properties to be suitable for printing, whilst simultaneously being able to cross-link/polymerise following deposition. Recently, we have also seen the application of this fabrication method with decellularized organ matrices, typically used as an alternative to this method, to produce bioinks with more relevant biochemical cues from the original tissue and also a suitable ratio of ECM proteins, something that is a challenge from a formulation perspective^73,74^. Achieving suitable, tissue-like mechanical properties in the resulting constructs is also the focus of research efforts, as many soft matrices are not able to hold their shape and weight.

The translation of bioprinting for applications in regenerative medicine during space exploration also has several mission-specific challenges. Whilst 3D printing offers the obvious advantages in terms of automation, it is also susceptible to malfunctioning, as with many equipment types. More importantly however, are the considerations regarding stability and integrity of potential implants during printing within a reduced gravity environment, particularly when trying to reproduce complex anatomies. Recent studies have been trying to simulate this limitation by employing extrusion-based printers in an inverted fashion, against Earth’s gravity^67^.

A bioprinting facility was specifically implemented on-orbit (2019) for the purpose of printing tissue-like mimics in support of producing eventual organ-like analogues in space - the Techshot Inc. - nScrypt/BioFabrication Facility (BFF)^34^, and most recently, an upgrade to BFF developed with the aim of printing human-like tissue mimics, announced in November 2022^75^. The latter offers a better temperature control in the system, one of the factors important for consistency in bioinks. For a review of additional bioprinters on the ISS see ref. ^67^. The results from this recently installed on-orbit bioprinting facility for early tissue-like prototypes are yet to be released, however it is widely believed that manufacturing 3D tissue and organ-like parts in a reduced gravity environment will prevent the collapse of structures and sedimentation normally taking place on Earth. While this may be true for the microgravity environment, the partial gravity levels on the Moon and Mars might be sufficient to create similar technical difficulties. Therefore, further research is needed to confirm this matter.

Additionally, biofabricating hard tissues presents further difficulties, as bone, for example, contains multiple phases (organic, inorganic and cellular, embedded in the mineral component) which are interconnected in a highly organised manner down to the micron and nano scales. Therefore, achieving that organisation between a calcium phosphate cement and a suitable bioink, while also preserving the viability of the mineral-phase cells is a major challenge in generating bioprinted, functional bone replacements.

The difficulties reported with soft tissues stem from the fact that low viscosity bioinks have to be used for production, which requires the final constructs to undergo additional culturing and several levels of conditioning post-printing. Currently, the biofabrication facilities described above benefit from an additional adjacent facility, the Advanced Space Experiment Processor (ADSEP), where additional treatment can take place.

At the same time, when trying to manufacture complex (multi-phase) or large-scale tissues in a challenging gravitational environment, both hard and soft tissues would, however, require some form of support matrix to prevent movement and the failure of structures during the process, particularly at interfaces. This is particularly relevant for the manufacturing of gradients seen in all tissues, such as those found in skin layers or the bone-ligament/tendon. This scaffolding would also address other factors that could be detrimental, such as the extended printing times associated with large or complex tissues, which could lead to structural changes in the print or a decrease in viability.

A solution to these limitations in long term might be printing the required construct suspended within a support matrix, such as a polymeric fluid gel bath (Fig. 5). These non-Newtonian gels which are increasingly used in tissue engineering, behave as fluids under mechanical stress and as solids in the absence of this force, making them ideal for use in reduced gravity. Therefore, they can greatly aid with traditional biofabrication by acting as a temporary, biocompatible supports and provisionally allowing the movement of the extrusion needle through the matrix at the same time as self-healing around the print. The tissue or organ-like part can subsequently be recovered non-destructively from the bath following polymerisation (e.g. induced chemically). Many of these fluid baths can be generated from reagents such as polysaccharides already widely used in laboratory applications for generating hydrogel scaffolds in tissue engineering, such as agarose^76,77^, agar (composed of agarose and agaropectin)^78,79^ and alginate^80,81^ which have good biocompatibility and gel forming properties. These alternative formulations produced through shear cooling of hot solutions throughout the sol-gel transition, leads to a ‘fluid-gel’ matrix. These can be used for providing structural support rather than a culture template, to aid with suspending a second hydrogel containing cells, thus supporting it during the cross-linking process whilst being able to self-heal immediately. A potential application of this process in an operational context is described below.

## The production of tissues analogues in reduced gravity using suspended manufacture

**Fig. 6:**
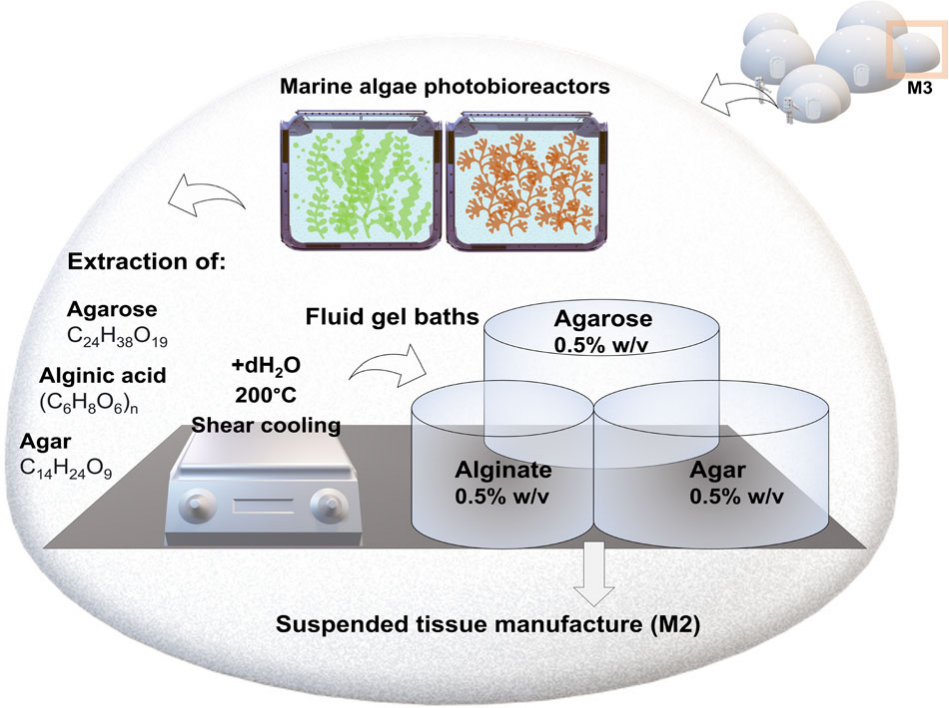
The materials bioprocessing module. An auxiliary module would be necessary for material bioprocessing and extraction of raw biochemical components. The requirement to produce cellular/acellular tissue substitutes of varying geometries in a reduced gravity environment using 3D printing will likely necessitate a supporting matrix to prevent movement and contain the construct during printing. Fluid gel baths can be used for this purpose, to act as a suspension matrix during printing, thus preventing construct failure during the process. These scaffolds can be easily reconstituted from powdered polysaccharides derived from different algal species. These algal masses can be cultivated in already existing photobioreactors on-site and processed in this module for the extraction of alginate, agar or agarose, which can then be reconstituted using basic steps (dissolved in water at high temperatures and cooled under shear). The resulting fluid gel baths would facilitate needle movement during the extrusion process, acting as fluids during the deposition process of biological material whilst self-healing around the print. These can be subsequently stored in sterile containers until use and transferred to the tissue engineering module where they can be used for the suspended layer manufacture of the desired construct.

Polymeric fluid baths are generated from hydrophilic polysaccharides extracted from the cell walls of marine algal species, dissolved in water at high temperatures and subsequently cooled under shear^82,83^. Low concentrations (as low as 0.5% w/v) are required to generate these suspending baths for tissue engineering applications and therefore a small quantity of these lightweight powders is required for the gelation process, making it feasible to carry several kilograms of these raw materials across a long-distance mission. Alternatively, in the long-term, these can be produced on-site, since the extraction processes of these polysaccharides from algae are chemically straightforward (involving limited steps and equipment) and well characterised (involving mechanical processing, separation and chemical treatment)^84^. Furthermore, because algae have been heavily considered as a dietary source as they may be essential for meeting nutritional demands in a deep-space mission, algal bioreactors are likely to be a part of future mission cargo, to provide a nutritional biomass^85^ but also for other life-support requirements, such as converting carbon dioxide into oxygen, with one such prototype (a photobioreactor) having been already tested on the ISS^86^. Therefore, it would be feasible to incorporate already existing marine photobioreactors within an auxiliary biomaterials processing unit in the habitat to extract key polysaccharides on-site, including agarose (C_24_H_38_O_19_), agar (C_14_H_24_O_9_) or alginic acid (C_6_H_8_O_6_)_n_. A description of this process is illustrated in Fig. 6.

In the long-term, the generation of raw materials on site from multipurpose technologies would decrease the mission costs and would provide a solution to the volume and mass constraints which limit the amount of supplies that can be launched for long-duration or long-distance missions.

## Using planetary resources for a sustainable laboratory infrastructure

As expected, a large proportion of this equipment would have to be transported from Earth. Due to space and significant costs, it would be unfeasible to transport all the required materials with human crews/manned spacecrafts and these could be instead launched to the planetary surfaces and landed to sites in the regions of interest, where they can be assembled after crew arrival. Such sites would be, as with normal habitat requirements, located in the proximity of water resources and have a good exposure to sunlight which would be essential for solar power generation. However, the Moon and Mars in particular contain an abundance of additional resources that could support such a habitat.

## CO_2_ processing options for tissue engineering

The abundance of CO_2_ on Mars is likely to make this resource valuable for a significant number of habitat operations and as such, it is very likely that a large proportion of the initial equipment on-site would be conversion equipment. Culturing cells within mammalian incubators requires an atmosphere of 5–7% CO_2_ to maintain a stable physiological pH through a CO_2_-bicarbonate based buffer system in the growth media. On Earth, this is achieved through storage of this gas inside large cylinders, which are connected to incubators that source the gas through tubing and inject small quantities within the growth chamber based on feedback. Carrying supplies of CO_2_ to Mars would be neither possible nor necessary as this planet contains an abundance of this gas in the atmosphere, which is composed of approximately 95% CO_2_. This means that storing this gas in large quantities will not be necessary and it could instead be gradually captured/extracted and stored as required.

## Sterilisation

The renewable energy generated by solar panels, in combination with the abundance of CO_2_ available can also be used for the conversion of this chemical to ethanol under copper-containing catalysts, as described recently^87,88^, which would be beneficial to generate sterilisation reagents that are required for disinfection, not only for tissue engineering, medical procedures and biohazardous waste destruction, but also for general maintenance (preventing the growth of microorganisms).

## Processing into plastic consumables

Recently, methods have been described for converting CO_2_ to ethylene using an electrochemical method^89,90^. Ethylene is widely used in the chemical industry and in polymeric form, as polyethylene, it is the most common plastic used in manufacture. This chemical could therefore be further processed on-site into much needed kits, including plastic-based laboratory consumables, which are often used in large quantities.

## Low-temperature storage

Tissue engineering reagents, medical supplies including therapeutics, as well as materials traditionally used in implantology, including haemostatic agents, typically require storage at low temperatures to preserve function and prevent degradation. The need for additional refrigeration equipment would create further operational challenges, not only because of the costs associated with sending and transporting heavy cargo through space, but also because this equipment has to be custom built and modified for operating within a reduced gravity environment, as the typical air movement/currents are not distributed similarly as seen in the Earth instruments.

However, unlike Earth, both the Martian and Lunar surfaces have very low temperatures depending on the geographical location^43,91^. Temperatures on Mars average approximately −53° Celsius globally^91^, while the Moon harbours some of the coldest sites in the solar system^43^). This resource may represent a useful and cost-effective way of storing temperature-sensitive materials in a contained manner without energy consumption.

## Cryostorage

One of the most important considerations for banking biological material (e.g. cells/tissues) on a planetary station and ensuring long time viability is the access to deep cryopreservation resources. This can be technically achieved theoretically, as Mars, for example, has seasonal polar caps which contain an abundance of frozen carbon dioxide (dry ice)^92^, also a common cryostorage agent in the biomedical industry, which offers further advantages, such as the ease of transportation. As discussed above, sourcing according to seasonal planetary phenomena will be key to this process. While the north polar region is covered with CO_2_ ice during winter, the south cap is covered throughout the entire year with CO_2_ frost and it is thought that the water ice form is also present within or underneath these deposits^91^. Frozen CO_2_ can therefore be extracted for long-term preservation of cell material brought from Earth, at ultra-low temperatures (i.e. approximately −80° Celsius), as well as to maintain any enzymatically-critical biological agents at an equivalent temperature as intended on Earth. The energy input required for storage within a biobank habitat would be minimal, as dry ice can be stored in lightweight containers such as polystyrene boxes and can be refilled when required. Furthermore, these containers can be collapsible, providing a reduction in storage requirements. Due to the volatile nature of dry ice (and the substantial exchange of CO_2_ between the atmosphere and the polar caps), throughout the year or alternatively, during the spring/summer seasons, its transition into the gaseous form of CO_2_ can be exploited for use with additional culture equipment, such as the cell incubating devices discussed above. It was previously estimated that the mass of CO_2_ sublimed from the south polar cap is equal or larger than 7.9 × 10^12^ metric tons^93^.

## Surgical module/Operating theatre design considerations

**Fig. 7:**
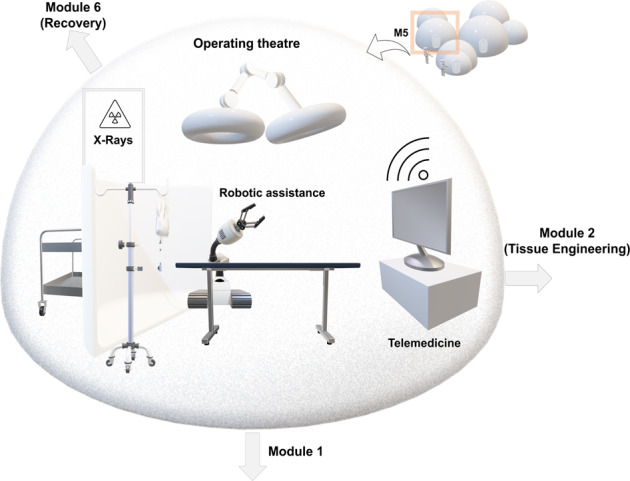
Set-up of a surgical module, containing an operating theatre. The surgical module would be connected to the tissue/bio-engineering module to allow a quick transfer of engineered (a)cellular substitutes/autologous grafts to the theatre and of minor tissue explants for incubation within the tissue engineering facilities. In addition to the key surgical apparatus, this module would benefit from imaging devices (e.g. X-Ray), essential in evaluating fractures and implant positioning. Audio-visual equipment, such as large monitors, would be essential for situations where telemedicine is an option and remote terrestrial support in real time is feasible. Robotic assistance may play a supporting role in surgery, particularly in delivering tools and ensuring fluid containment.

Performing surgery in reduced gravity would require careful design of the physical environment (Fig. 7). Orthopaedic trauma surgery in particular requires the application of percussive impulses to implants, sustained force to displaced bones or dislocated joints, and torque to screws used to hold implants and bone fragments in place. The reactive forces acting upon the surgeon would lead to their acceleration in the opposite direction (or counter-rotation in the case of torque), a situation analogous to astronauts engaged in mechanical activity during extra-vehicular activity. Therefore, surgical instrument and operation theatre design would have to be modified from their Earth-based counterparts to allow for the neutralisation of these forces.

## Imaging modalities

Intra-operative X-rays are often essential to confirm the position of implants or bones. The risk of this ionising radiation to those in close proximity to the patient (surgeons, anaesthetists etc) is mitigated by the use of high atomic-weight shielding in flexible gowns and within the X-ray machine, all of which comes with prohibitively high mass that would be costly to get into orbit or beyond. Lightweight design, low atomic-weight materials, and the impracticality of increasing the distance between the source and the occupants in habitat enclosures would exacerbate this problem. Reduction in scatter (collimation), combined with the use of in-situ resources (such as regolith between elements of a habitat) might be key in initial stages. Alternatively, the use of ultrasonographic technology might offer a radiation-free, more flexible solution. This technology has already been considered for diagnosis in trauma scenarios during space missions and has been tested within the LEO environment of the International Space Station, particularly to address the use by crewmembers/non-physicians with minimal sonography training, with the addition of remote guidance. In these studies, it has been shown to be useful for monitoring cervical^94^, thoracic^95^, lumbar and sacral regions^96^ for anatomical changes in microgravity. It has also been evaluated as having a good level of diagnostic accuracy for detecting limb fractures in a recent systematic review/meta-analysis^97^. Ultrasonography offers the advantage of compactness, portability and minimal space requirements and could, therefore, be operated successfully by non-medical staff in a hazardous environment.

## Containment of tissue fluid

Management of traumatic or surgically-induced bleeding would require further design considerations in the reduced microgravity environment. On Earth, blood pools according to gravity and can obscure the surgical field. This is managed through a combination of suction applied via a flexible tube or via absorbent swabs. In micro- and reduced gravity, blood may become free floating, which would constitute less of a problem in terms of obscuring the surgeon’s view but would carry risks of biological hazard to others and contamination of the habitat infrastructure. Absorbent swabs and suction may play a role but engineering solutions may also be required, such as a flow of air over the operative field associated with a capture device to collect any stray blood or fluids.

## (Bio)Medical scenarios and prioritisation of activities

**Fig. 8:**
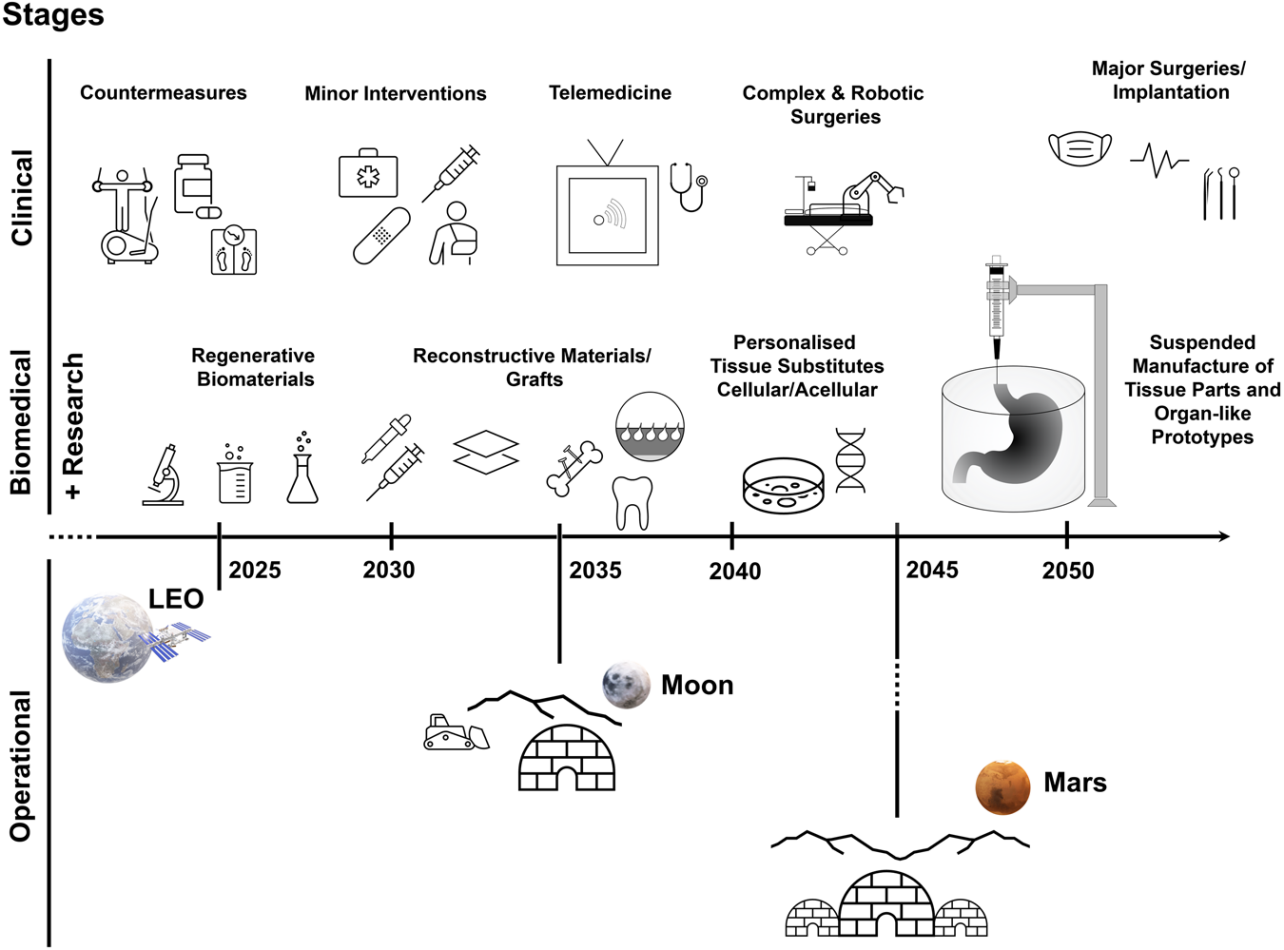
A timeline of the required (bio)medical activities within planetary habitats during the next decades. Implementation of a life support infrastructure will require a coordinated biomedical and medical activity on-site, during multiple phases of increasing complexity, to mitigate the risks associated with the operational demands. It is very likely that the next decade, dominated by the construction stages of a lunar base, will require bio(medical) assistance to be present in the form of regenerative biomaterials, patches for skin damage repair, injectable systems and acellular therapies, followed by skeletal stabilisation and xenograft/autograft/allograft interventions in the late 2030s–early 2040s through established facilities and with telemedical and remote assistance. With further development, personalised tissue engineered constructs (similar to current commercial ones) and over the next decades, more complex tissue and organ-like parts might become a viable option within the late 2040s, and 2050s, respectively, at the same time as the foundations are created for an equivalent Mars base.

Figure 8 provides an overview of the required biomedical and clinical activities within planetary habitats and the timeframes for potential implementation within a mission scenario based on operational demands and also their current level of development. Implementation of a life support infrastructure to ensure crew health, a sustained presence on the planetary surface and mission continuity will require a coordinated biomedical and clinical activity on-site, during multiple phases of increasing complexity, to mitigate the risks associated with the hazardous environments and operational tasks.

It is likely that in the short-term (2030–2035), during the initial phases of lunar base development, traditional damage mitigation methodologies will be required to manage emergencies in this isolated environment, involving formulation and preparation of acellular regenerative biomaterials, injectable systems, casting, manual mixing of suitable matrices, fillers and patches for skin repair. Emergency medical interventions are likely to involve orthopaedic stabilisation materials such as fixation screws or sutures and are likely to be assisted through telemedicine.

In subsequent years, with the technological diversification of facilities, complex tissue reconstruction could become a viable option. Surgical reconstruction will be likely be focused on isolating and transferring autografts to the injured sites, as well as small-medium engineered autografts which can be cultured in the tissue engineering facility, and would be similar to existing FDA-approved therapeutics such as cultured epidermal autografts (Epicell®^46,98^, measuring approximately 50 cm^2^ and used for treating deep dermal burns), or the autologous cultured chondrocytes on porcine collagen membranes (MACI®) for fixing cartilage defects.

Orthopaedic and maxillofacial surgeries on site are likely to involve traditional metallic materials (e.g. hip repair), ceramics and as well as acellular, physically-treated scaffolds (or xenografts) with native geometry and biochemistry (late 2030s–2040s).

With further developments in tissue engineering, bioreactors for tissue perfusion and 3D biofabrication technologies over the subsequent years, multi-phase, personalised tissues and ultimately, organ-like parts could be generated in an isolated environment (2040s–2050s+), at the same time as the foundations are created for an equivalent Mars base. These procedures would concomitantly require the presence of complex robotic and well-equipped clinical facilities on site, which will allow surgeries of higher complexity (in late 2040s) and major surgeries (2045–2050s).

At the same time, there are additional matters of a regulatory and translational nature which will be associated with the production of artificial tissues and organ-like parts for human application, which will need to be implemented in the manufacturing sites of the habitat in early stages and which stretch beyond factors like containment and ensuring maximal sterility. This is because the acellular tissue replacement constructs will likely be categorised as class II medical devices, whereas the cell-containing scaffolds will probably receive a class III medical device classification^98^, which will require significant on-site validation and performance testing. As such, these final products will need to undergo rigorous assessment for quality assurance and process monitoring. The tissue engineering facility is therefore likely to play an additional, strategic role in routine testing of biocompatibility, biomaterial stability and validation, which can be conducted in a personalised manner.

## Outlook and summary

The return to the Moon and the multi-part journey to Mars will require a significant number of resources, with the most important being undoubtedly the human crews. Manned missions to these distant planetary bodies with challenging geologies and environmental conditions will pose a high risk of tissue damage and medical emergencies. Furthermore, tissue degradation induced by the change in gravitational conditions during the extended transfer journey and on arrival to a reduced gravity planetary environment further complicates the matter and increases the risk of musculo-skeletal damage even during moderate intensity tasks. These clinical contexts will have to be managed autonomously on-site and as such, habitat enclosures allowing both tissue substitute generation and implantation will be key for ensuring astronaut health and therefore a sustainable presence on these planets. In this perspective article, we proposed a modular-type habitat structure that would support such operations, namely tissue substitute development (cellular and acellular), implantation and recovery. These considerations were based on experience with wet lab operational requirements as well as clinical practice.

It is likely that in the short-term, traditional tissue reconstruction methodologies will be used to manage emergencies in deep-space (2030–2035), involving casting and hand mixing of suitable matrices, which are easier to implement in a spaceflight scenario. Surgical reconstruction options will be likely be initially focused around isolating and transferring minor autografts to the injured site as well as acellular, physically-treated scaffolds with native geometry and biochemistry (throughout 2040s). With further development of tissue engineering and 3D biofabrication technologies over the following years, more complex, personalised tissues and ultimately organ-like structures could be generated in an isolated environment (2040s–2050s+). However, the ability to produce constructs which can maintain integrity in a planetary hypogravity setting is essential to this process, hence why support matrices will be required, such as fluid gel baths, from which the constructs can subsequently be recovered. Operating theatre and surgical tool design will also have to be assessed and adapted to the challenging gravitational conditions of these planets. Aspects such as fluid containment kit, restraining equipment to prevent patient falling/drifting during operation, surgical tool adaptation and integration of X-ray/Ultrasonographic kit for assessing outcomes might be critical. Finally, processing of local resources will be key for generating laboratory and medical infrastructure. The Moon and Mars in particular, contain an abundance of resources in multiple forms, which can be extracted or converted into useful products to generate laboratory-specific reagents and tools. Over the next years, it will be essential to generate further predictions on these matters, which will ultimately dictate the design of these space settlements and the technology required to support these.

## Data Availability

The data supporting the findings of this study is available within the paper.
